# Early Development and the Functional Correlation of Brain Structural Connectivity in Preterm-Born Infants

**DOI:** 10.3389/fnins.2022.949230

**Published:** 2022-07-05

**Authors:** Tingting Liu, Zhiyong Zhao, Yuqing You, Fusheng Gao, Ying Lv, Mingyan Li, Chai Ji, Can Lai, Hongxi Zhang, Dan Wu

**Affiliations:** ^1^Key Laboratory for Biomedical Engineering of Ministry of Education, Department of Biomedical Engineering, College of Biomedical Engineering and Instrument Science, Zhejiang University, Hangzhou, China; ^2^Department of Radiology, Children’s Hospital, Zhejiang University School of Medicine, Hangzhou, China; ^3^Department of Child Health, The Children’s Hospital, Zhejiang University School of Medicine, Hangzhou, China

**Keywords:** structural network, diffusion MRI, tractography, infant brain development, language outcome

## Abstract

Exuberant axon growth and competitive pruning lead to dramatic and comprehensive changes in white matter pathways of the infant brain during the first few postnatal months, yet the development of structural configuration in early infancy has not been fully characterized. This study aimed to investigate the developmental trajectory of structural connectivity reflecting relative fiber density in 43 preterm-born infants aged 0–3 months of corrected age without any complications utilizing probabilistic tractography based on fiber orientation distribution and to explore the potential function correlation associated with the network properties based on the Chinese Communication Development of Infant at 10 months of corrected age. The findings revealed significant increases in global efficiency, local efficiency, normalized clustering coefficient, and small-worldness (*p*_adj_ < 0.001 for each), while the normalized characteristic path length showed a non-significant decrease with age (*p*_adj_ = 0.118). Furthermore, those findings were validated by another parcelation strategy. In addition, the early local efficiency was found to be significantly correlated with words understood at 10 months of corrected age. A unique developmental pattern of structural networks with enhancing efficiency and the small-world property was found in early infancy, which was different from those of neonates or toddlers. In addition, this study revealed a significant correlation between local efficiency and late language comprehension, which indicated that enhanced structural connectivity may lay the structural foundation for language specialization.

## Introduction

Exuberant axon growth and competitive pruning lead to dramatic and comprehensive changes in white matter pathways of the infant brain during the first few postnatal months (Innocenti and Price, 2005; Vanderhaeghen and Cheng, 2010), which may induce a profound alteration in the topology of white matter. Infancy is regarded to be the fastest stage of global and local reconfiguration (Cao et al., 2017), but is not yet fully characterized.

Structural network with diffusion MRI-based tractography provides a powerful approach to investigate the brain connectivity, which has been used to study the network dynamics during the perinatal period and childhood (Hagmann et al., 2010; Brown et al., 2014; van den Heuvel et al., 2015; Baum et al., 2017), and reveals that neonates, children, and adults exhibit a distinct development pattern of brain structural connectivity. Previous studies revealed that the clustering coefficient and small-worldness increased during the perinatal period (Brown et al., 2014; van den Heuvel et al., 2015; Batalle et al., 2017) and then decreased from toddler to adult (Hagmann et al., 2010; Dennis et al., 2013). Combined with the results of another study, which revealed a significant decrease in clustering coefficients and small-worldness from neonates to childhood (Huang et al., 2015), it is reasonable to speculate that there is an inflection point in network properties during infancy.

However, the developmental pattern of brain structural networks in infancy remains less understood, possibly due to the lack of data and difficulty of acquisition in this period. Yap et al. (2011) conducted a longitudinal study to investigate the developmental trends of white matter connectivity in 39 participants at 2 weeks, 1 year, and 2 years and found that all brains exhibited small-world properties with increasing local efficiency. Tymofiyeva et al. (2013) performed deterministic tractography in 8 preterm-born neonates, 8 term-born neonates, 10 six-month-old infants, and 7 adults and found that the clustering coefficient decreased from preterm-born neonates to 6-month-old infants and then increased until adult; while the small-worldness increased from preterm-born neonates to term-born neonates, kept steady until 6 months old, and then decreased until adult. These two studies with small sample sizes did not reach consistent conclusions, and the discrete scan timepoints (neonate, 6 months, and 1 year) hindered the continuous characterization of development. In addition, tensor-based tractography used in the previous studies was not ideal for immature infant brain due to difficulties in distinguishing whether the signal reduction was due to immature tissue or crossed fibers within voxels (Descoteaux, 1999), which can be addressed by the probabilistic tractography method based on fiber orientation distribution (FOD; Tournier et al., 2010).

Moreover, structural brain networks at an early stage may indicate the functional outcome at a later stage. Baum et al. (2017) revealed the mediation effect of modular segregation of structural networks on the development of executive function in youth. However, the functional correlates of early white matter networks were still unknown. This study aimed to investigate the development of structural brain networks in 0–3 months old preterm-born infants utilizing high angular resolution diffusion imaging (HARDI) and the potential function under network measures by correlating with 10 months old Chinese Communication Development of Infant (CDI).

## Materials and Methods

### Subjects

In total, sixty-seven preterm-born infants were enrolled for MRI scans from 0 to 3 months of corrected age at the Children’s Hospital of Zhejiang University School of Medicine. The parents of all participants provided written informed consent, and the ethical approval was granted by the institutional review board of the local hospital. Exclusion criteria included (1) extremely preterm birth (less than 28 weeks); (2) any acquired lesions on MRI (assessed by a radiologist T.L.); (3) visible artifacts on MRI; (4) congenital malformation or syndrome; (5) encephalopathy caused by various factors; (6) intrauterine growth restriction; (7) intracranial infection; (8) alcohol or an illicit drug during pregnancy; and (9) neurological or psychiatric family history. Among the 67 infants recruited, the following infants were excluded: 16 participants who failed the CDI follow-up, 3 extremely preterm-born infants (GA at birth less than 28 weeks), 1 participant with visual brain parenchymal lesion, 2 participants with intrauterine infection, and 2 participants with poor image quality.

### Image Acquisition

A certified nurse gave all infants 50 mg/kg oral or enema chloral hydrate 30 min before the scan. Ear protectors were applied for hearing protection, and a vacuum immobilization mat was applied to reduce motion. The scanner’s physiological monitoring system was used to continuously monitor the heart and respiratory rates by a neonatologist in the scanner room. MRI was carried out on a 3T Philips Achieva system with an 8-channel head coil. A single-shot echo-planar imaging sequence was used for acquiring multi-shell HARDI data, and 32 non-collinear diffusion-encoding directions were acquired with *b*-values of 800 and 1,500 s/mm^2^. An additional b0 image with the opposite phase-encoding direction was acquired for eddy correction. The particularized parameters were as follows: TR/TE = 9,652/115 ms, voxel size = 1.5 mm × 1.5 mm × 2 mm, FOV = 180 mm × 180 mm × 120 mm, and SENSE acceleration factor of 2.

### Data Preprocessing

First, the HARDI data were preprocessed using MRtrix3,^1^ including denoising (Veraart et al., 2016), Gibb’s ring artifact removal (Kellner et al., 2016), eddy correction (Andersson and Sotiropoulos, 2016), slice-to-volume correction (Andersson et al., 2017), and bias correction (Tustison et al., 2010). The weighted linear least squares method (Basser et al., 1994) was used for obtaining fraction anisotropy (FA) and mean diffusivity (MD).

### Cortical Parcelation

The Edinburgh Neonatal Atlas 50 (ENA50) (Blesa et al., 2020) with multiple templates (e.g., DTI metrics) and parcelation schemes was transformed to the individual subject space utilizing a multi-channel registration method based on MD, FA, and the mean DWI contrasts (Djamanakova et al., 2013). Two sets of parcelation schemes including the University of North Carolina (UNC; Shi et al., 2011) and Melbourne Children’s Regional Infant Brain (M-CRIB; Alexander et al., 2017) parcellations were obtained for each participant. The transformation matrix between ENA alignment to individual data was used to transform the ENA gray matter probability map to individual space, and the voxels with gray matter probability less than 0.4, as well as subcortical and cerebellar regions, were excluded, and 78 cortical regions of UNC were defined as structural network nodes to study network dynamics. In addition, for assessing whether findings were biased by the parcelation schemes, 68 cortical nodes from the M-CRIB atlas were used for validating the reproducibility of the results.

### Whole-Brain Tractography

The individual FOD was generated from the preprocessed HARDI data according to the MRtrix3 multi-shell multi-tissue constrained spherical deconvolution (MSMT-CSD) pipeline (Jeurissen et al., 2014), and all individual data were up-sampled to an isotropic voxel of 1 mm. Then, the whole-brain tractography was performed based on individual FOD images utilizing the second-order integration over the FOD method (Tournier et al., 2010), and the tractography profile was as follows: step size = 0.5 mm, minimum/maximum length = 10/250 mm, maximum angle = 90°, and cutoff = 0.05. Afterward, 10 million streamlines were filtered to 1 million utilizing the spherical-deconvolution informed filtering of tractograms (SIFT) method (Smith et al., 2013), which selectively removed streamlines such that the streamline density was as close as possible to fiber density.

### Network Construction

**FIGURE 1:**
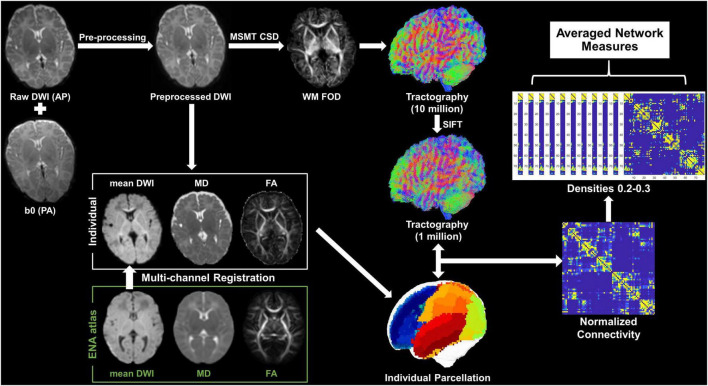
Pipeline of data analysis. Mean DWI, MD, and FA were generated from the preprocessed dMRI and were then co-registered with the ENA atlas to obtain individual parcelation. The WM FOD was calculated according to the MSMT-CSD pipeline in MRtrix3, based on which whole-brain tractography was obtained followed by a SIFT operation. The individual parcelation and filtered tractography were used to generate a 78 × 78 symmetric connectivity matrix. Network measures were calculated at a series of network densities, and then, they were averaged for statistical analysis.

The number of fiber tracts connecting two cortical nodes was first normalized to node volumes (sum of two nodes) and then normalized to the total number of tracts across the brain. The tract density between each pair of the 78 cortical nodes forms a 78 × 78 symmetric connectivity matrix. Concerning false-negative connections due to spurious streamlines in probabilistic tractography, we set the network density (the proportion of actual connections among potential connections) to range from 0.2 to 0.3 with an interval of 0.01 (11 thresholds) to calculate network measures, where 0.2 is the minimal network density with full connectivity, and then averaged the measures at all densities (Figure 1). In addition, the procedure was repeated based on the M-CRIB parcelation to evaluate the reproducibility of the findings from UNC parcelation.

### Network Measures

Five global network measures were calculated for each infant: local efficiency, global efficiency, normalized clustering coefficient, normalized characteristic path length, and small-worldness.

The global efficiency (*E*_glob_) is the average of the inverse shortest path length in a structural network (Latora and Marchiori, 2001), which quantifies the exchange of information across the whole network where information is concurrently exchanged, and can be calculated as follows:


(1) $$E_{g\,l\,o\,b}=\frac{1}{n}\,\sum_{i\in N}\frac{\sum_{j\in N,j\neq i}{\left(d_{i\,j}^{w}\right)}^{-1}}{n-1}$$

where $d_{i\,j}^{w}$ is the shortest weighted path length between *i* and *j*.

The local efficiency (*E*_*loc*_) is the global efficiency computed on the neighborhood of the node (Latora and Marchiori, 2001), which quantifies a network’s resistance to failure on a small scale, and can be calculated as follows:


(2) $$E_{l\,o\,c}=\frac{1}{2}\,\sum_{i\in N}\frac{\sum_{j,h\in N,j\neq i}{\left(w_{i\,j}\,w_{i\,h}\,{\left[d_{j\,h}^{w}\,\left(N_{i}\right)\right]}^{-1}\right)}^{1/3}}{k_{i}\,\left(k_{i}-1\right)}$$

where *w*_*ij*_ is the connection weight between node *i* and *j*; $d_{j\,h}^{w}\,\left(N_{i}\right)$ is the shortest path length between *j* and *h* (node *i* is the only neighbor of *j* and *h* along this shortest path); and *k*_*i*_ is the number of links connected to node *i*.

The weighted clustering coefficient (CC) is the proportion of triangles around a single node, and thus, the average CC of the network reflects the prevalence of clustered connections (Watts and Strogatz, 1998). In this study, a variant CC was calculated, which is free from the disproportionate influence of low-degree nodes (Newman, 2003):


(3) $$\text{CC}=\frac{\sum_{i\in N}2\,t_{i}^{w}}{\sum_{i\in N}k_{i}\,\left(k_{i}-1\right)}$$

where $t_{i}^{w}$ is weighted geometric mean of triangles around node *i* and can be calculated as follows:


(4) $$t_{i}^{w}=\frac{1}{2}\,\sum_{j,h\in N}{\left(w_{i\,j}\,w_{i\,h}\,w_{j\,h}\right)}^{1/3}$$

The characteristic path length (CPL) is the average shortest path length among all node pairs in the network and measures the network integration (Watts and Strogatz, 1998), and it can be calculated as follows:


(5) $$d_{i\,j}^{w}=\sum_{a_{u\,v}\in g_{i↔j}^{w}}f\,\left(w_{u\,v}\right)$$

where *f* is a mapping from weight to length and $g_{i↔j}^{w}$ is the shortest weighted path between node *i* and *j*.

Small-worldness characterizes a network that is remarkably more clustered than a random network, but has roughly the same CPL as a random network, and can be calculated as follows:


(6) $$\text{S}=\frac{C\,C/C\,C_{\text{rand}}}{C\,P\,L/C\,P\,L_{\text{rand}}}$$

where *CC*/*CC*_rand_ is the normalized CC of a random network and *CPL*/*CPL*_rand_ is the normalized CPL of a random network.

### Statistical Analysis

All statistical tests were performed utilizing the R-Project 4.1.2.^2^ Factors that were potentially associated with network measures were first screened using univariate analysis, and the significant ones were taken as covariates into the multiple regression to analyze the correlations between network measures and gestational age (GA) at birth, and postmenstrual age (PMA) at scan. Pearson’s correlation analysis was conducted between network measures obtained from UNC and M-CRIB parcelation to evaluate the effect of the parcelation scheme.

For assessing the correlation between network properties and language outcomes, univariate analysis was used to identify potential factors that were significantly associated with the Chinese CDI scores, which were included as covariates for subsequent multiple analysis. It was noted that the observed network measures were first regressed against PMA at scan and bodyweight at scan, and then, the residuals were used as PMA-corrected indicators of network for subsequent language correlation analysis. The *p*-values were adjusted by the false discovery rate method. The significance level for all analyses was set at 0.05.

## Results

### Demographic Information of Participants

**FIGURE 2:**
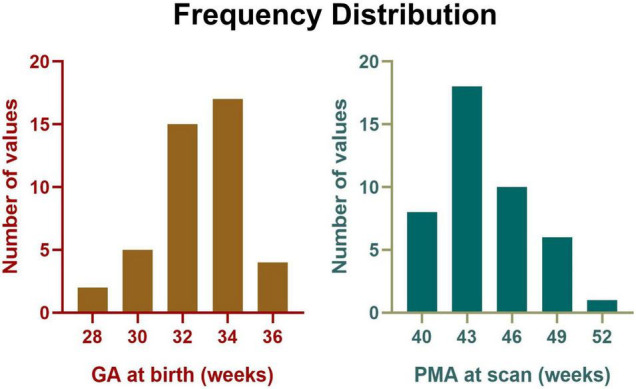
Frequency distribution of GA at birth and PMA at scan for all participants.

After exclusion, 43 preterm-born infants aged 39.9–50.9 postmenstrual weeks (0–3 months of corrected age) without any complications were included in the final analysis, with GA at birth ranging from 28.1 to 35.6 postmenstrual weeks (detailed information was demonstrated in Figure 2 and Table 1). In addition, the mean head motion was 0.65 mm, which showed no significant effect on the network measures based on an ANCOVA.

**TABLE 1:** Demographic information of the infants and their communication development of infant (CDI) sub-scores at 10 months.

Demographic information	
Gender (male/female)	25/18
Gestational age at birth (weeks)	32.7 ± 1.9
Postmenstrual age at scan (weeks)	44.4 ± 2.7
Multiple-birth/singleton	1.83 ± 0.4
Head motion (mm)	0.64 ± 0.3
Body weight at birth (kg)	42.3 ± 4.2
Body length at birth (cm)	11/32
Delivery (vaginal/cesarean)	4.50 ± 0.9
Body weight at scan (kg)	30.9 ± 3.6
Maternal age at delivery (years)	6.1 ± 1.5
Maternal education	25/18
**Chinese CDI (10 months of corrected age)**	
Words understood	38.4 ± 24.3
Words production	1.1 ± 1.6
Phrases understood	2.0 ± 1.6
Actions and gestures	4.3 ± 2.4

### No Significant Effect of Gestational Age Was Found on Network Measures

In this study, PMA at scan, bodyweight at scan, and delivery were found to be significantly correlated with network measures and were treated as covariates in the multiple regression analysis between network properties and GA at birth. However, no significant correlation was found, indicating that the network measures were not affected by preterm birth in this study.

### Developmental Trajectory of Structural Brain Network in 0–3-Month-Old Infants

**FIGURE 3:**
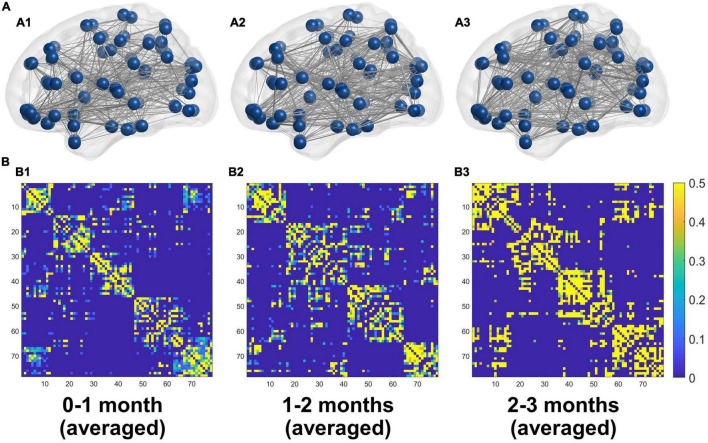
**(A)** 3D representations of the mean structural networks of 0–1 (A1), 1–2 (A2), and 2–3 (A3) months groups. **(B)** The averaged structural connectivity matrices of 0–1 (B1), 1–2 (B2), and 2–3 (B3) months groups.

**FIGURE 4:**
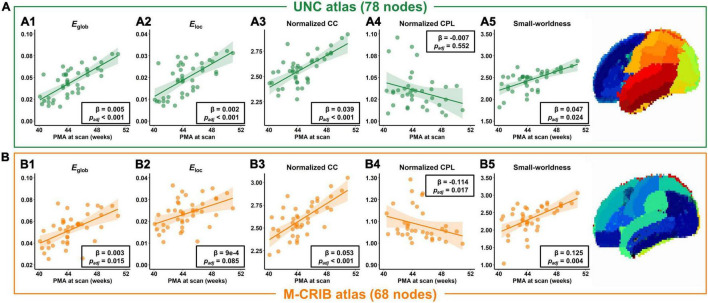
Developmental trajectories of network measures in infants aged 0–3 months, in terms of the global efficiency (*E*_glob_), local efficiency (*E*_loc_), normalized clustering coefficient (CC), normalized characteristic path length (CPL), and small-worldness, based on the UNC atlas **(A)** or M-CRIB atlas **(B)**.

The averaged structural connectivity matrices of infants aged 0–1, 1–2, and 2–3 months (Figure 3) demonstrated an enhancing fiber density with age. All brain network measures except the normalized CPL exhibited significant age-dependent alterations in infants aged 0–3 months (Figure 4A). These findings revealed that both integration (*E*_glob_) and segregation (*E*_loc_ and normalized CC) increased significantly with age, and the developmental rates (β) of *E*_glob_, *E*_loc_, and normalized CC were 0.005, 0.002, and 0.039 per week, respectively (*p*_adj_ < 0.001 for each). The normalized CPL showed a gradual decline with age, although the statistic was not significant (β = −0.007 per week, *p*_adj_ = 0.552). Furthermore, the increased clustering and decreased CPL together resulted in the fast growth of small-worldness (β = 0.047 per week, *p*_adj_ = 0.024) (Figure 4A).

In addition, the network properties obtained from M-CRIB parcelation revealed a similar developmental trajectory to that of UNC parcelation (Figure 4B), and the developmental trajectories of the two parcellations were significantly correlated (Figure 4 and Table 2).

**TABLE 2:** Correlation between the network measures from University of North Carolina (UNC) and Melbourne children’s regional infant brain (M-CRIB) atlases.

Network measures	Correlation coefficient	*P*-value
*E*_glob_	0.582	**<0.001**
*E*_loc_	0.600	**<0.001**
Normalized CC	0.807	**<0.001**
Normalized CPL	0.556	**<0.001**
Small-worldness	0.831	**<0.001**

*Bold value represents adjusted p-value < 0.05.*

### Local Efficiency Was Significantly Correlated With Words Understood at 10 Months

**FIGURE 5:**
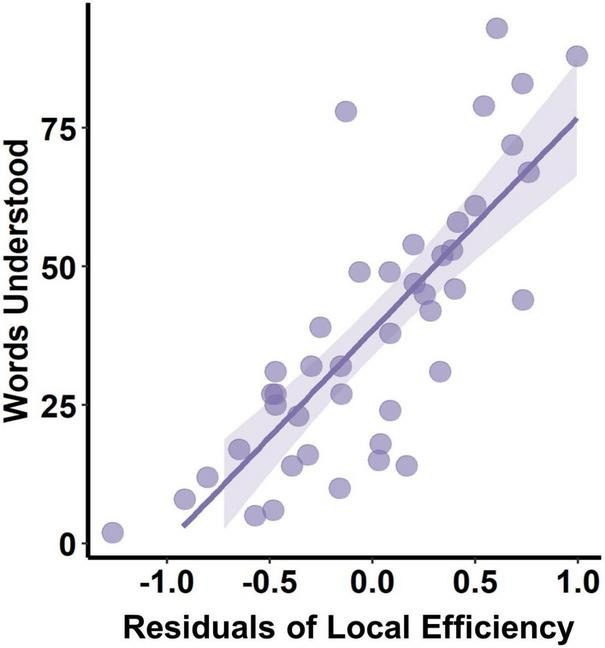
Correlation between PMA-corrected local efficiency and words understood at 10 months.

The PMA at scan, body weight at scan, delivery, and maternal education were found significantly correlated with communication outcome at 10 months of corrected age and were used as covariates in multiple regression between the network measure and CDI scores. However, only the local efficiency was found significantly correlated with words understood (correlation coefficient = 0.339, *p*_adj_ = 0.047) (Figure 5), while no other network measures were found to be significantly correlated with communication outcomes (Table 3).

**TABLE 3:** Correlation of postmenstrual age (PMA)-corrected network measures and communication outcomes, and the correlation coefficients were demonstrated.

Residuals of network measures	Words understood	Phrase understood	Words production	Actions and gestures
*E*_*glob*_	0.008	−0.185	0.103	−0.191
*E*_*loc*_	**0.339**	0.161	0.063	0.136
Normalized CC	0.137	−0.077	0.062	−0.138
Normalized CPL	0.136	0.190	0.028	0.193
Small-worldness	0.086	0.004	0.011	−0.270

*Bold value represents adjusted p-value < 0.05.*

## Discussion

This study investigated the developmental trends of structural brain networks in infants aged 0–3 months, which was not presented before. The structural networks were generated utilizing a probabilistic tractography based on FOD followed by a SIFT, and the findings were validated by another parcelation. The results also indicated that higher local efficiency in early infancy may be associated with language comprehension at a later stage.

### Enhanced Information Transfer Efficiency Within the Infant Brain

This study revealed a significant and monotonic increase in global and local efficiency in infants aged 0–3 months, which indicated an enhanced information transfer efficiency and greater fault tolerance within the infant brain. Few studies have discussed the evolution of network efficiency in infants and have not reached consistent conclusions. Batalle et al. (2017) investigated the development of structural brain networks in neonates aged 25–45 postmenstrual weeks and revealed an increase in global efficiency but a decrease in local efficiency with age. However, all studies in infancy used selected scan times and found inconsistent findings. For example, Huang et al. (2015) revealed that neonates had significantly lower structural brain network efficiency than toddlers, while Yap et al. (2011) reported a significant increase in local efficiency in 1-year-old toddlers compared with neonates, but global efficiency remained constant. The results of this study were consistent with Huang et al. and provided a continuous developmental profile of network efficiency during early infancy.

### Linear Rising Clustering Coefficients and Small-World Properties

This study found significantly higher normalized CC and small-world properties of structural brain network in infants aged 0–3 months and a slight decrease in normalized CPL. This finding was consistent with the results of network development in perinatal studies utilizing DTI (Brown et al., 2014; van den Heuvel et al., 2015; Batalle et al., 2017), but in contrast to those studies comparing newborns and toddlers (Huang et al., 2015). This implied that the small-world property of the brain structural network may not monotonically decrease during infancy, and there is likely a turning point of small-world properties between 3 and 12 months, which remains to be confirmed by a wider age range of infant studies.

### Higher Local Efficiency Was Associated With Better Words Understood

After controlling for age, we found that higher local efficiency of structural connectivity at birth was associated with better word comprehension at 10 months, and this is the first report on the relationship between structural connectivity and cognition development to the best of our knowledge. Local efficiency measures the capability of a network to information transmission locally, indicating how well each cluster exchanges information when the index node is eliminated (Latora and Marchiori, 2001). In addition, the local functional connectivity of the network is thought to be the foundation for functional segregation and specialization (Sporns, 2013), which may be associated with cognitive functions. For example, Chan et al. (2014) studied the functional network organization across the lifespan (20–89 years) utilizing functional MRI and revealed that segregation was predictable for long-term memory function. Given that functional network organization is determined by the underlying structural network (Cao et al., 2017), we speculate that enhanced structural connectivity in local regions related to language may lay the structural foundation for language specialization.

### Limitation

Several limitations of this study should be noted. First, this study lack term-born infants as healthy control. We excluded infants whose brain development might be compromised by certain factors, e.g., extreme preterm birth and encephalopathy, to ensure that relatively healthy participants were enrolled. In addition, the fact that there was no correlation between GA at birth and the structural network measurements supported that our study population was not affected by preterm-born. Nevertheless, future validation of the current findings from healthy term-born infants would be ideal. Second, participants were sedated with chloral hydrate. However, chloral hydrate is recognized as safe and is often applied to infants for minimizing head motion (Finnemore et al., 2014). Despite that sedation may reduce brain activity (Williams et al., 2015), there is no evidence that it might affect infant brain structure. Finally, due to the narrow age range of participants in this study, the potential inflection point of the small-world property was not revealed, and future studies with a larger age range were needed.

## Conclusion

This study investigated the developmental trajectories of structural connectivity reflecting relative fiber connections in infants aged 0–3 months, and the findings were validated by another parcelation scheme. A unique developmental pattern of structural networks with enhancing efficiency and small-world property was found in early infancy. In addition, this study revealed a significant correlation between local efficiency and late language comprehension, which indicated that enhanced structural connectivity in local regions related to language may lay the structural foundation for language specialization.

## Data Availability Statement

The raw data supporting the conclusions of this article will be made available by the authors, without undue reservation.

## Ethics Statement

The studies involving human participants were reviewed and approved by the Children’s Hospital of Zhejiang University School of Medicine. Written informed consent to participate in this study was provided by the participants’ legal guardian/next of kin.

## Author Contributions

TL contributed to the data processing and manuscript writing. ZZ contributed to the conception of the study. YY, FG, HZ, and CL contributed to MRI scanning. YL, ML, and CJ contributed to the participants recruitment. DW contributed to the conception and design of the study and major revision of manuscript. All authors contributed to the article and approved the submitted version.

## Conflict of Interest

The authors declare that the research was conducted in the absence of any commercial or financial relationships that could be construed as a potential conflict of interest.

## Publisher’s Note

All claims expressed in this article are solely those of the authors and do not necessarily represent those of their affiliated organizations, or those of the publisher, the editors and the reviewers. Any product that may be evaluated in this article, or claim that may be made by its manufacturer, is not guaranteed or endorsed by the publisher.

## Footnotes

1
https://www.mrtrix.org/

2
https://www.r-project.org/
